# Asymmetric efficacy of VNS within a single patient with bilateral focal frontal lobe epilepsy: A case report

**DOI:** 10.1007/s00701-025-06653-x

**Published:** 2025-08-26

**Authors:** Masanobu Kumon, Shunsuke Nakae, Daijiro Kojima, Noeru Kawase, Yuichi Hirose

**Affiliations:** Department of Neurosurgery, Fujita Health University Hospital School of Medicine, Aichi, Japan

**Keywords:** Vagus nerve stimulation, Drug-resistant epilepsy, Lateralized efficacy, Stereo-electroencephalography

## Abstract

The lateralized efficacy of vagus nerve stimulation (VNS) remains insufficiently explored. We report a case of drug-resistant epilepsy with bilateral frontal lobe seizure onset, treated with left cervical VNS. Preoperative video- electroencephalogram revealed predominant interictal discharges in the right hemisphere and frequent seizures from both hemispheres. Following VNS, overall seizure frequency decreased. Notably, stereo-electroencephalography performed 15 months postoperatively showed a marked reduction in right-sided seizures, while left-sided seizures remained frequent. This case highlights the potential lateralized effect of VNS in a single patient with bilateral frontal lobe epilepsy, suggesting that VNS may preferentially suppress seizures originating from the right hemisphere.

## Introduction

Vagus nerve stimulation (VNS) is a surgical option for drug-resistant epilepsy (DRE), with its efficacy supported by multiple studies [17, 18]. It is believed to suppress seizures by modulating thalamic and limbic networks via vagal afferent pathways [14]. The procedure involves placing electrodes around the cervical vagus nerve and implanting a subcutaneous pulse generator [8].

VNS is typically applied to the left vagus nerve [15], which may give rise to the hypothesi**s** that seizure lateralization and network localization may influence therapeutic outcomes. However, evidence regarding the impact of seizure focus laterality on VNS efficacy remains limited. To our knowledge, the only report suggesting such a relationship is by Hödl et al. [6].

In this study, we report a case of a patient with independently originating frontal lobe seizures who underwent VNS therapy. Postoperative stereoelectroencephalography (SEEG) enabled detailed lateralized seizure analysis, revealing a marked reduction in seizures originating from the right hemisphere.

### Case presentation

The patient was a man in his 40 s. At 16, he underwent tumor resection for a craniopharyngioma using a right frontal approach. Postoperatively, he received 50 Gy of whole-brain radiation therapy. Seizures began following the surgery, and despite continuous antiseizure medication (ASM), he developed DRE.

At 45, he underwent tumor resection of the left frontal lobe. Pathology revealed an oligodendroglioma, IDH-mutant and 1p/19q co-deleted, grade 3. He received six cycles of temozolomide chemotherapy. Over time, seizure frequency increased, prompting referral to our institution for further management.

**Fig. 1:**
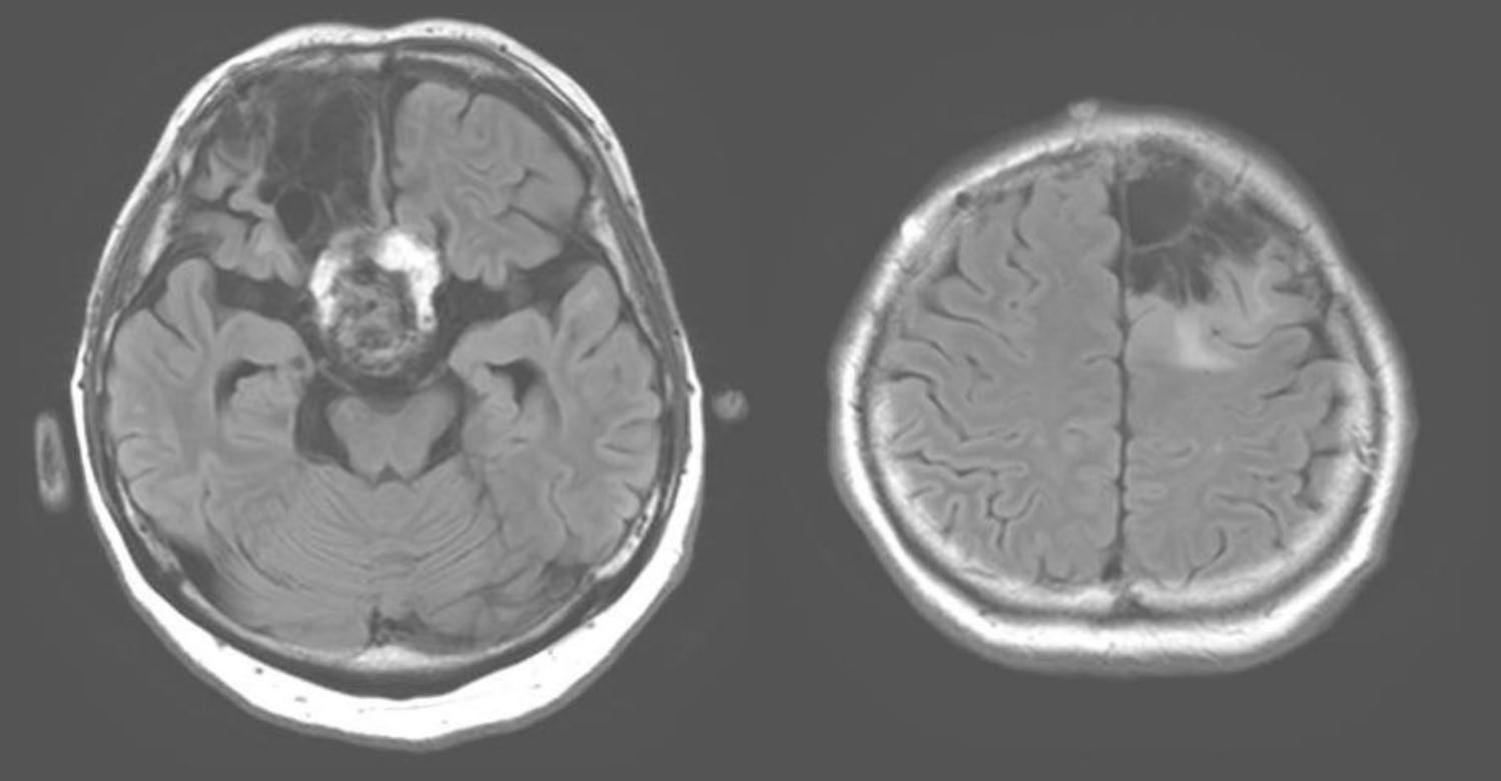
Preoperative FLAIR MRI of the patient. A residual craniopharyngioma lesion and encephalomalacia were observed in the right orbitofrontal region. In the left frontal lobe, postoperative changes and a residual oligodendroglioma lesion were identified along the margin of the resection cavity

At the time of presentation, he was taking 400 mg of carbamazepine and 1000 mg of levetiracetam, but seizure control was inadequate. He experienced daily focal aware seizures (FAS) characterized by sensorimotor symptoms in the left upper limb, as well as focal to bilateral tonic–clonic seizures with impaired awareness. Magnetic resonance imaging (MRI) revealed residual craniopharyngioma and encephalomalacia in the right frontal lobe, and a postoperative cavity in the left frontal lobe consistent with prior glioma resection (Fig. 1). Interictal electroencephalogram (EEG) showed frequent spike-and-wave discharges predominantly at F4.

**Fig. 2:**
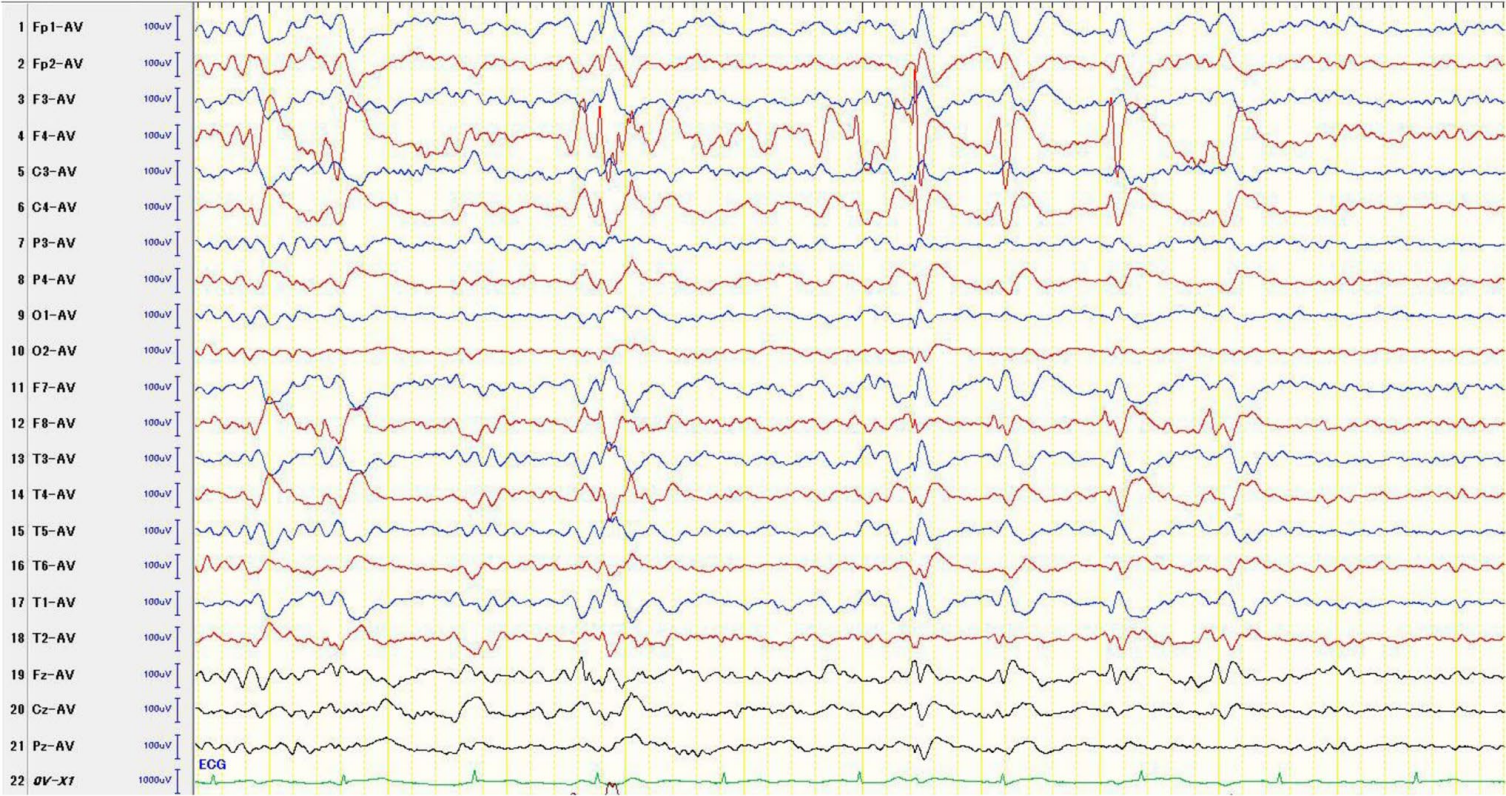
Interictal scalp EEG findings during video monitoring. Prominent spike-and-wave discharges were observed predominantly in the right hemisphere, centered at electrode F4

Although adjustments in ASMs led to partial improvement, refractory seizures continued despite triple therapy. Therefore, long-term video-EEG (VEEG) monitoring was conducted over a two-day period. During this time, six focal impaired awareness seizures (FIAS) were captured. Among these, two involved head turning to the left, suggesting onset in the right hemisphere; three showed rightward turning, indicating onset in the left hemisphere; and one was non-lateralizing. Interictal discharges were more prominent in the right hemisphere (Fig. 2). Seizures appeared to originate with similar frequency from both hemispheres.

VNS was selected based on bilateral seizure onsets on VEEG and the presence of a WHO grade 3 oligodendroglioma, presumed to be radiation-induced. Following implantation, seizure frequency decreased, improving to about one FAS per month. However, gradual posterior expansion of Fluid-attenuated Inversion Recovery (FLAIR) hyperintensity around the resection cavity and recurrence of presumed left-sided FIAS led to the decision to pursue resection of both the tumor and epileptogenic focus.

**Fig. 3:**
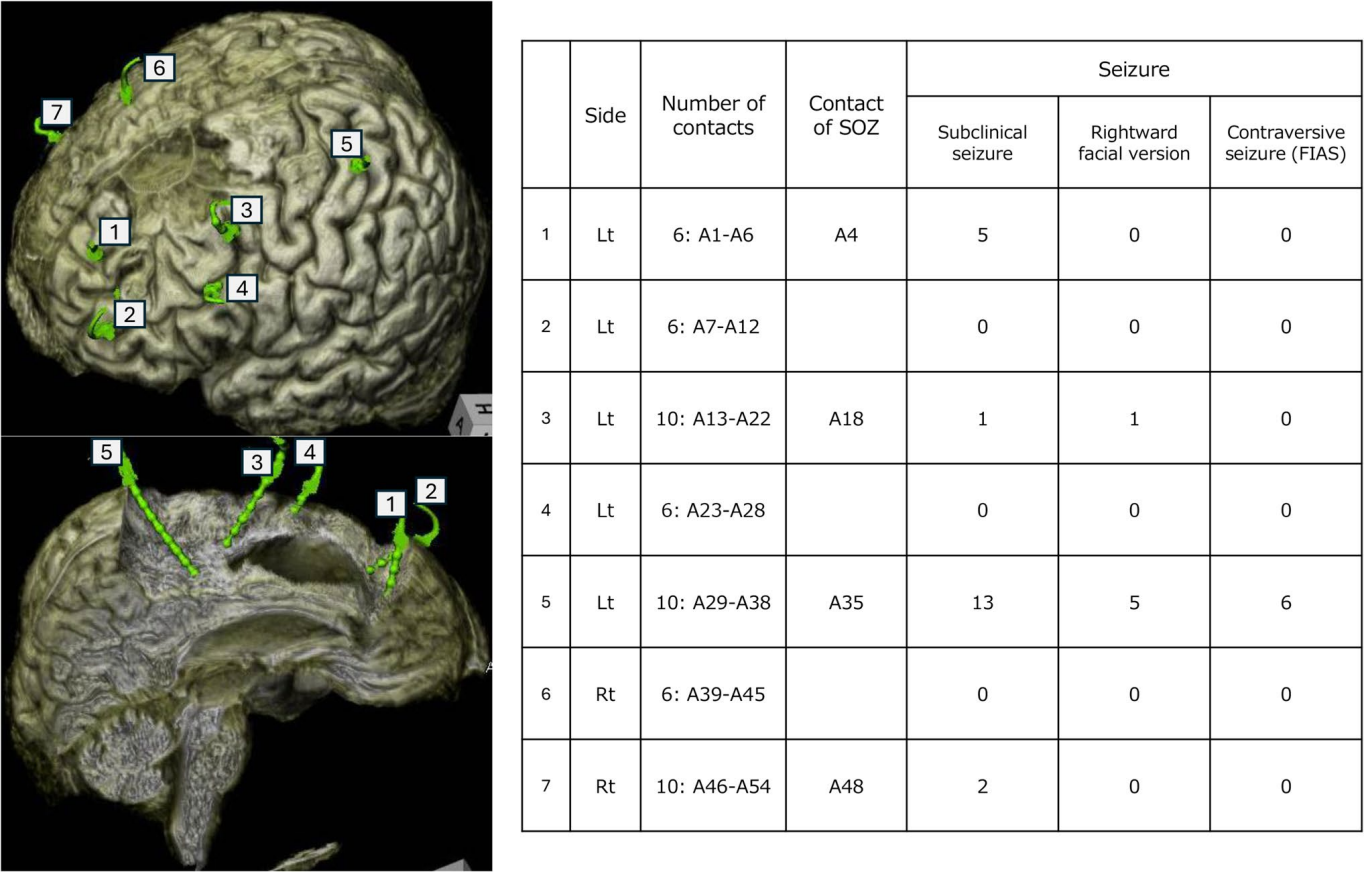
Fused image of preoperative 3D MRI and post-SEEG insertion plain Computed tomography, along with a summary of recorded seizures. The numbers in the image indicate electrode channel numbers

One year and three months after VNS implantation, SEEG was performed. A total of seven depth electrodes with 54 contacts were implanted: five surrounding the left frontal resection cavity and two surrounding the right frontal encephalomalacia (Fig. 3). All ASMs and VNS stimulation were temporarily discontinued to observe seizure activity.

**Fig. 4:**
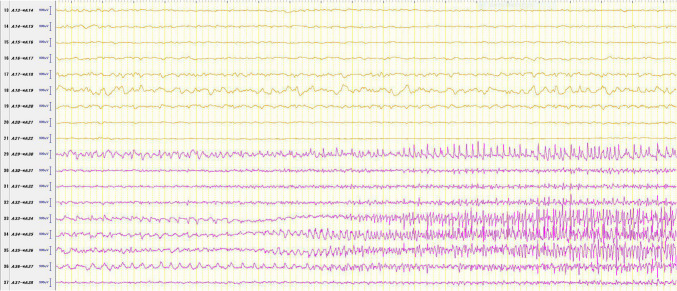
SEEG findings during ictal events. All recorded focal impaired awareness seizures (FIAS) originated from contact A35

Over three days, a total of 31 subclinical seizures originating from the left frontal lobe and right versive seizures were observed (Fig. 4). In contrast, only two subclinical seizures from the right hemisphere were recorded, indicating a markedly lower seizure frequency on that side. A distribution of seizure onset zones is summarized in Fig. 3.

Two weeks after SEEG, the patient underwent awake surgery for tumor resection and epileptogenic zone removal. No new neurological deficits occurred, and he was discharged home. No seizures were reported postoperatively, corresponding to an Engel class I outcome.

## Discussion

This study describes a patient with DRE with bilateral frontal lobe foci who underwent VNS. SEEG performed 15 months post-VNS revealed a marked reduction in seizures originating from the right hemisphere. To the best of our knowledge, this is the first study to detail seizure laterality using SEEG post-VNS implantation in a patient with bilateral epileptic foci, providing valuable clinical insight.

In this case, careful treatment planning was essential due to the patient’s complex medical history and bilateral epileptic foci. The strategy was based on initial video-EEG findings showing seizure onsets in both frontal lobes, as well as the presence of a WHO grade 3 oligodendroglioma in the left frontal lobe. Extensive right frontal resection could complicate future surgery for the left tumor. Therefore, VNS was selected as the first surgical intervention. As the tumor later expanded posteriorly, tumor resection became necessary. To achieve both seizure reduction and oncological control, SEEG was conducted to evaluate the required extent of resection. Extensive electrodes were implanted in the left frontal lobe surrounding the tumor, while only two electrodes were placed in the right frontal lobe. If seizure activity had been more frequent on the right, resection of the tumor alone would have been considered sufficient. Although MRI showed mild left hippocampal atrophy, hippocampal electrodes were omitted since combined resection with the left frontal lobe was not feasible.

The distribution of efferent vagus nerve fibers is asymmetrical, and right-sided stimulation carries a higher risk of bradyarrhythmia [19]. Right-sided VNS may also delay therapeutic effects, making left-sided VNS generally preferred [16]. VNS responsiveness may vary with seizure lateralization; one previous study has reported that patients with right-sided foci may show better outcomes than those with left-sided foci [6]. In this case, FIAS frequency was similar bilaterally before VNS but became left-lateralized after, suggesting better seizure control in the right hemisphere, as reported by Hödl et al.

The vagus afferent network (VagAN), a central autonomic network, mediates the effects of VNS via projections from the nucleus tractus solitarius to brainstem nuclei such as the locus coeruleus, and to other regions including the hypothalamus [5]. In pediatric patients with DRE, VNS responsiveness has been associated with strong functional connectivity in the left insula and temporal lobe, suggesting that impaired VagAN function in the left hemisphere may underlie VNS non-responsiveness [7]. In the present case, it is possible that left frontal lobe pathology disrupted this network, contributing to the poorer seizure control on the left side.

The insula receives afferent input from the vagus nerve and plays a key role in autonomic regulation. Some studies have reported that the left insula predominantly mediates parasympathetic control, while the right insula is more involved in sympathetic regulation [2], suggesting that this difference may contribute to the lateralized effects of VNS. Moreover, stronger connectivity between the insula and anterior cingulate cortex has been suggested in the right hemisphere [11], raising the possibility that left-sided VNS modulated right frontal lobe excitability through insular pathways in this case. These findings underscore the likelihood of lateralized effects of VNS due to anatomical and functional network asymmetries.

An alternative explanation for the seizure laterality post-VNS involves differences in etiology. Tumor histology is known to influence the intractability of epilepsy; patients with malignant tumors often show limited response to VNS therapy [12]. However, some studies suggest that tumor progression may be more predictive of VNS responsiveness [12]. In this case, seizures from the right frontal lobe may have been related to surgical corridor injury, whereas the left frontal seizures were caused by an oligodendroglioma. This disparity in etiology may have contributed to the observed asymmetry in VNS efficacy.

Although oligodendrogliomas typically have a favorable prognosis among adult-type diffuse gliomas [10], radiation-induced malignant gliomas are often associated with poor outcomes [4]. Seizures are common in glioma patients, with a higher incidence in low-grade tumors [9]. VNS has shown efficacy in brain tumor–related epilepsy, with long-term seizure suppression comparable to that seen in non-tumor epilepsy [13]. Furthermore, VNS may be effective regardless of prior tumor resection or focal excision [3]. Nevertheless, tumor progression may weaken the therapeutic effect of VNS, and the differing etiologies in this case may have influenced the variable response.

Focal seizures depend on specific neural networks and understanding how VNS modulates them is key to outcome prediction. In bilateral epilepsy, network dynamics differ from unilateral cases, making such cases useful for studying whole-brain effects. Frontal lobe epilepsy generally has better outcomes than temporal lobe epilepsy [1], so comprehensive evaluation is essential in bilateral cases.

This study is limited by the short duration of pre-VNS scalp VEEG monitoring, which may have affected lateralization accuracy. In addition, this study has the limitation that intracranial EEG evaluation was not performed both before and after VNS implantation; therefore, the laterality of seizure onset and the precise frequency of seizures originating from each hemisphere could not be definitively determined. Nevertheless, pre-VNS scalp VEEG demonstrated independent foci in both hemispheres with a comparable seizure frequency. Given that multiple tumors were involved and that the treatment strategy addressed both drug-resistant epilepsy and recurrent radiation-induced grade 3 glioma, our approach was designed to achieve not only seizure control but also tumor control. This approach appears clinically justified, as both seizure and tumor control have been favorable to date. However, right-sided discharges and contraversive seizures seen before VNS were largely absent in post-VNS SEEG, suggesting VNS-induced persistent suppression of right-sided seizures.

## Conclusion

This case demonstrates that in a patient with bilateral independent foci, VNS may be more effective for right-sided foci. These findings suggest a potential strategy: resection for the left focus and VNS for the right in patients with bilateral foci and similar seizure frequency. However, as the underlying etiologies differed between hemispheres, further studies are needed to validate this approach.

## Data Availability

No datasets were generated or analysed during the current study.
